# A role for nucleotides in support of breast cancer angiogenesis: heterologous receptor signalling

**DOI:** 10.1038/bjc.2011.134

**Published:** 2011-04-19

**Authors:** N Yokdang, J D Tellez, H Tian, J Norvell, S H Barsky, M Valencik, I L O Buxton

**Affiliations:** 1 Department of Pharmacology, Centre for Molecular Medicine, University of Nevada School of Medicine, Mail Stop 573, Reno, NV 89557, USA; 2 Department of Pathology, University of Nevada School of Medicine, Reno, NV 89557, USA; 3 Department of Biochemistry, University of Nevada School of Medicine, Reno, NV 89557, USA

**Keywords:** breast cancer, dormancy, NDPK, nucleotides, purinergic receptors, signalling, angiogenesis

## Abstract

**Background:**

Human breast carcinoma cells secrete an adenosine 5′-diphosphate transphosphorylase (sNDPK) known to induce endothelial cell tubulogenesis in a P2Y receptor-dependent manner. We examined sNDPK secretion and its effects on human endothelial cells.

**Methods:**

Nucleoside diphosphate kinase (NDPK) secretion was measured by western blot and enzyme-linked immunosorbent assay, while transphosphorylase activity was measured using the luciferin-luciferase ATP assay. Activation of MAPK was determined by western blot analysis, immunofluorescence and endothelial cell proliferation and migration.

**Results:**

A panel of breast cancer cell lines with origin as ductal carcinoma, adenocarcinoma or medullary carcinoma, secrete sNDPK-A/B. Addition of purified NDPK-B to endothelial cultures activated VEGFR-2 and Erk_1/2_, both of which were blocked by inhibitors of NDPK and P2Y receptors. Activation of VEGFR-2 and ErK_1/2_ by 2-methylthio-ATP (2MeS-ATP) was blocked by pretreatment with the P2Y_1_-specific antagonist MRS2179, the proto-oncogene non-receptor tyrosine kinase (*Src*) inhibitor PP2 or the VEGFR-2 antagonist SU1498. Nucleoside diphosphate kinase-B stimulates cell growth and migration in a concentration-dependent manner comparable to the effect of vascular endothelial growth factor. Treatment of endothelial cells with either NDPK-B or 2MeS-ATP induced migration, blocked by P2Y_1_, *Src* or VEGFR-2 antagonists.

**Conclusion:**

sNDPK supports angiogenesis. Understanding the mechanism of action of sNDPK and P2Y_1_ nucleotide signalling in metastasis and angiogenesis represent new therapeutic targets for anti-angiogenic therapies to benefit patients.

Vascular P2 nucleotide receptors activated by ATP include both ligand-gated ion channels (P2X) and hetero-trimeric G protein-coupled (P2Y) receptors (White and Burnstock, 2006). P2Y receptors are recognised as important regulators of carcinogenesis and endothelial regulation and are integral modulators of platelet aggregation and blood flow regulation (Buxton *et al*, 2001; Burnstock, 2006; White and Burnstock, 2006). Extracellular ATP activates P2Y receptors on vascular endothelial cells to release vasoactive mediators such as nitric oxide, prostacyclin and additional ATP (Yang *et al*, 1994; Buxton *et al*, 2001), which elicit vasodilation and propagate this effect downstream (Buxton *et al*, 2001; Kashiwagi *et al*, 2005). We have proposed that breast tumour cells that secrete nucleoside diphosphate kinase (NDPK) promote their intravasation and extravasation from blood vessels by generating nucleotides to activate P2Y receptors on endothelium. Furthermore, we and others (Seye *et al*, 2004; Rumjahn *et al*, 2009) have shown that P2Y_1/2_ receptors activated by extracellular ATP transactivate VEGFR-2, demonstrating a direct link between extracellular nucleotide regulation and the VEGFR-2 signalling cascade in the service of angiogenesis.

Nucleoside diphosphate kinase domains are present in a large family of structurally and functionally conserved proteins from bacteria to humans. There are eight isoforms of this plurifunctional protein, NM23-H1-H8 (McDermott *et al*, 2008). NM23-H1 and NM23-H2 (NDPK-A and NDPK-B) are the most abundant and have significant roles in normal and diseased sates (Postel, 1998; Hartsough and Steeg, 2000). In non-transformed cells, NDPK functions as an NDPK regenerating ATP levels for intracellular ‘*housekeeping*’ enzymes by covalently transferring the *γ*-phosphate from a nucleoside triphosphate (NTP) such as GTP to a nucleoside diphosphate acceptor (NDP; e.g., ADP). Nucleoside diphosphate kinase has also been shown to act as a histidine kinase, transcription activator and an exonuclease (Postel, 2003). NM23 was originally described as non-metastatic gene 23, which was found in mouse carcinoma cells and was thought to be inversely related to metastasis (Postel, 2003; Palmieri *et al*, 2006) although this has been controversial. There is evidence to support an intracellular role for NM23/NDPK in tumour metastasis (Hamby *et al*, 2000; Roymans *et al*, 2002), and high tissue levels of NDPK-A protein have been found in patients with breast carcinoma (Heimann *et al*, 1998; Sauer *et al*, 1998). Nucleoside diphosphate kinases are secreted by various solid and haematological malignancies (Anzinger *et al*, 2001; Niitsu *et al*, 2001; Okabe-Kado *et al*, 2005a) and promote growth of acute myelogenous leukaemia cells (Okabe-Kado *et al*, 2009b) and endothelial cells *in vitro* (Rumjahn *et al*, 2007). Extracellular NDPK activity regulates extracellular nucleotide levels on the cell surface in many cell types (Yang *et al*, 1994; Lazarowski *et al*, 2000; Spychala *et al*, 2004). We have shown that membrane bound ecto-NDPK acts extracellularly to contribute to the maintenance of vascular tone regulating blood flow *via* nucleotide receptor activation (Buxton *et al*, 2001). Nucleoside diphosphate kinase regulation of blood flow first leads us to propose a pathological role for secreted NDPK (sNDPK) in cancer and tumour angiogenesis.

Here, we tested the notion that breast cancer cell lines, known to be metastatic *in vivo*, but not normal epithelial cell lines, secrete large amounts of extracellular NDPK-A/B resulting in primary endothelial cell migration and proliferation. Next, we determined the role of extracellular NDPK/ATP in the angiogenic pathway by using pharmacological targets that effect the exogenous mitogenic signalling from P2Y_1_R to mitogen-activated MAPK in endothelial cells. Importantly, we demonstrate that inhibition of P2Y_1_R, the tyrosine kinase phosphorylation activity of VEGFR-2, or the tyrosine kinase phosphorylation activity of the proto-oncogene non-receptor tyrosine kinase *Src*, prevents extracellular NDPK/2-methylthio-ATP (2MeS-ATP) from inducing ERK/MAP activation and migration. Understanding the mechanism of the sNDPK:P2Y signalling pathway may explain the failure of anti-angiogenic monotherapy in certain patients, and defines a new therapeutic target for future anti-angiogenesis treatment.

## Materials and methods

### Cell culture

Human breast cell lines HCC70, HCC202, HCC1143, MDA-MB-468, MDA-MB-231, MDA-MB-435, MDA-MB-156, MDA-MB-361 and MCF-7 as well as non-cancerous breast cells MCF-12 were obtained from ATCC (Rockville, MD, USA) and grown as recommended by the supplier. Primary cell lines of cloned *h*uman cord blood *e*ndothelial *c*olony forming cells (HEC) were purchased from Dynacell Life Sciences (Spring House, PA, USA) and used experimentally between passages 6 and 12. *H*uman cord blood *e*ndothelial *c*olony forming cells were grown in endothelial growth media-2 (EGM-2, Clonetics, Lonza, Basel, Switzerland) supplemented with 10 or 2% fetal bovine serum (FBS) (v/v), 1% penicillin–streptomycin (1000 U penicillin/1 mg streptomycin) and 0.2% fungizone (Sigma, St Louis, MO, USA). All cell lines were grown and maintained at 37°C in a humidified 5% CO_2_ atmosphere.

### Drugs and reagents

Yeast NDPK-B, MRS2179 (the P2Y_1_R antagonist), 2MeS-ATP (the P2Y_1_R agonist) and EA/EGCG (NDPK inhibitors) were obtained from Sigma. SU1498 (specific VEGFR-2 TRK inhibitor), suramin (the P2Y non-specific antagonist) and PP2 (*Src*-Tyr-*p* inhibitor) were obtained from Calbiochem (San Diego, CA, USA). Recombinant Nm23-h2 (NDPK-B) proteins were obtained from Abnova (Taipei City, Taiwan). PD098059 (ERK/MAPK inhibitor), VEGF_165_, anti-phosphotyrosine *p1175*, anti-mitogen-activated protein kinase *pERK*_1/2_ and mouse anti-MAPK (Erk_1/2_) were obtained from Cell Signaling Technology (Beverly, MA, USA) and mouse anti-GAPDH was from Santa Cruz Biotechnology (Santa Cruz, CA, USA).

### Preparation of sNDPK

All breast carcinoma cell lines and MCF-12 were grown to 80% confluence in T-150 tissue culture flasks, washed three times with PBS and conditioned media collected and concentrated as previously described (Rumjahn *et al*, 2007). Briefly, the extracellular fluid was concentrated for 30 min at 4°C and centrifuged at 2000 **g** using Amicon Ultra-15 10 kDa centrifugal filters (Millipore Corporation, Bedford, MA, USA). The concentrated conditioned fluid was analysed for sNDPK-B protein by immunoblotting and enzyme-linked immunosorbent assay (ELISA) and ATP production was compared with the activity of purified yeast NDPK-B. Human NDPK-B and yeast NDPK share 59% sequence identity at the protein level. Nucleoside diphosphate kinase domains are present in a large family of structurally and functionally conserved proteins from bacteria to humans that generally catalyse the transfer of *γ*-phosphates from an NTP such as GTP, to an NDP acceptor (e.g., ADP). Human NDPK-B contains 152 amino acids; the active site phosphohistidine intermediate is at residue 118. Yeast NDPK contains 153 amino acids; the active site of phosphohistidine intermediate is at residue 119 and the enzymatic site is identical to the human enzyme. While human NDPK-A and -B share 88% sequence identity at the protein level; the active sites and binding sites of these two isoforms are identical.

### Transphosphorylation activity

The conversion of ADP to ATP using GTP as the phosphoryl donor was quantified using the luciferin-luciferase ATP assay as previously described (Buxton *et al*, 2001). Briefly, partially purified sNDPK from the panel of breast cell lines and purified yeast NDPK-B (Sigma) employed as a positive control were incubated for 2 min with GTP (300 *μ*M) as a phosphoryl donor and ADP (30 *μ*M) as substrate. An equal volume of luciferin-luciferase ATP detection buffer (Sigma) was added and a single measurement of luminescence was recorded 10 s later on a Luminoskan luminometer (model RS, Labsystems, Helsinki, Finland). Relative luminescence units were adjusted for background and ATP conversion was measured against a standard curve, which was linear over three orders of magnitude.

### Indirect ELISA

Human sNDPK was detected by ELISA. Ninety-six well plates (Corning, Lowell, MA, USA) were coated with recombinant Nm23-h2 (NDPK-B) protein as standard sample (Abnova), or serum test proteins (experimental). Standard NDPK-B samples were employed over a range from 0.06 to 200 ng ml^−1^ in phosphate-buffered saline (containing 0.05% sodium azide); 50 *μ*l aliquots were added to wells and the plate incubated for 2 h at room temperature (RT) and then overnight at 4°C on a slow shaker. Blocking buffer (100 *μ*l) containing 5% BSA and Tween-20 (0.05%) in phosphate-buffered saline (PBST 0.05%) was added and incubated for 1.5 h at RT. Plates were then washed once with 150 *μ*l of PBST, incubated with primary mouse anti-nm23-H2 (NDPK-B) antibody (Abnova) diluted with PBST buffer containing 1 mM EDTA and 0.25% BSA (100 *μ*l) for 2 h at 37°C with slow rocking, and then washed three times with 150 *μ*l PBST. Plates were incubated with rabbit anti-mouse IgG horseradish peroxidase (HRP)-conjugate (Southern Biotech, Birmingham, AL, USA) for 2 h at 37°C with slow rocking and washed three times with PBST. Plates were developed by addition of 100 *μ*l of o-phenylenediamine dihydrochloride substrate (Sigma) and absorbance measured at 490 nm using a microplate reader.

### Immunoblotting of human sNDPK from breast cancer cell lines

To detect human sNDPK from conditioned fluid, the secreted proteins were resolved on SDS–PAGE gels and transferred onto nitrocellulose membranes and incubated at 4°C overnight with anti-NDPK-B mouse pAB (Abnova). Then, membranes were incubated with secondary antibodies conjugated to Alexa Fluor 680 (Invitrogen, Carlsbad, CA, USA) in 1 : 1 Odyssey blocking buffer (LI-COR Biosciences, Lincoln, NE, USA) and PBS with 0.1% Tween-20 (v/v). Bands were visualised using the Odyssey Infrared Imaging System (V2.04, LI-COR, Lincoln, NE, USA).

### Immunoblotting of proteins

Human cord blood endothelial colony forming cells were cultured to ∼75% confluence in EGM-2 with 10% FBS on 100 mm^2^ dishes and then switched to basal media (EBM-2) without growth factors supplemented with 2% FBS for 24 h. For combination treatment, HEC were pretreated with antagonists for 20 min before stimulation with agonists for another 10 min at 37°C. Antagonists were employed at concentrations chosen to provide maximal inhibition of the known target. PD098059, 50 *μ*M (IC_50_=2 × 10^−6^ M; Alessi *et al*, 1995), SU1498, 50 *μ*M (IC_50_=7 × 10^7^ M; Arbiser *et al*, 2000; Rollin *et al*, 2004), PP2, 100 nM (IC_50_=100 nM; Hanke *et al*, 1996; Karni *et al*, 2003), MRS2179, 10 *μ*M (*K*_B_=100 nM; Boyer *et al*, 1998; Baurand *et al*, 2001; Kaiser and Buxton, 2001, 2002) and suramin, 100 *μ*M (Beindl *et al*, 1996). The agonists employed were 100 ng ml^−1^ VEGF, 10 *μ*M 2MeS-ATP and 10 units of yeast NDPK-B (1 unit=0.76 *μ*g of NDPK-B converts 43.78 *μ*moles of ADP to ATP per min at 30°C). P2Y_1_ receptors are maximally stimulated by 10 *μ*M 2MeS-ATP (Ralevic and Burnstock, 1998; Rumjahn *et al*, 2007). The medium was removed and the plates rinsed twice in ice-cold PBS containing 0.5 mM Na_3_VO_4_ and suspended in lysis buffer containing 20 mM Tris HCl pH 8, 150 mM NaCl, 10% glycerol, 1% Triton X-100 or Nonidet P-40 (NP-40), 1 mM EGTA, 2.5 mM sodium pyrophosphate, 1 mM Na_3_VO_4_, 1 mM NaF and 1% Halt protease inhibitor (Pierce Biotechnology, Rockford, IL, USA). Adherent cells were scraped from the plates with a plastic cell scraper, collected in centrifuge tubes and incubated at 4°C on a rocker for 15 min. The cell lysate was centrifuged at 10 000 **g** at 4°C for 30 min, and supernatant protein concentration determined by Lowry assay. Proteins were resolved on SDS–PAGE gels and transferred onto nitrocellulose membranes and incubated at 4°C overnight with either rabbit anti-phosphotyrosine *p1175* or rabbit anti-mitogen-activated protein kinase *pERK*_1/2_. To detect total protein expression, membranes were incubated at 4°C overnight with mouse anti-MAPK (Erk_1/2_), mouse anti-GAPDH or mouse anti-VEGFR-2. Then, all of the proteins were incubated with secondary antibodies conjugated to either Alexa Fluor 680 or Alexa Fluor 800 fluorescent dye (Invitrogen). Bands were visualised using an Odyssey Infrared Imaging System (V2.04).

### Endothelial cell immunofluorescence assay

Human cord blood endothelial colony forming cells (5 × 10^3^) were plated in each well of an 8-well plate, grown to 75% confluence and incubated with EBM containing 2% FBS for 24 h before stimulation with antagonists for 20 min followed by agonist for another 10 min as described above. Each chamber was washed twice with sterile cold PBS and monolayers fixed with 4% paraformaldehyde in PBS for 15 min at RT. Each chamber was washed three times for 5 min with PBS, blocked (0.25% Triton-X, 5% donkey serum) for 60 min and then incubated with primary anti-*p*-ERK_1/2_ antibody containing 10% BSA and 0.25% Triton-X overnight at 4°C. Plates were then rinsed four times for 5 min in 1 × PBS–0.1% Tween (PBST) and incubated in fluorochrome-conjugated secondary antibody (donkey anti-rabbit Alexa 488) for 1 h at 4°C in the dark. Plates were subsequently washed with PBST four times for 5 min. The cells were stained with DAPI for 10 min at 4°C to reveal nuclei. The slides were washed three times for 5 min each in PBST and slide mounting solution was added and a coverslip was placed on top of the sections. Cells were viewed and imaged using the Olympus FluoView FV1000 confocal scanning microscope (Olympus, Center Valley, PA, USA).

### Endothelial cell proliferation assay

Human cord blood endothelial colony forming cells (7 × 10^3^) were seeded on 24-well plates, grown to 70% confluence, shifted to EBM-2 containing 2% FBS for 24 h and then treated with either 10 *μ*M ellagic acid (EA), 10 *μ*M epigallocatechin gallate (EGCG) with or without 2.5, 5, 10 or 20 units of NDPK-B (1 unit=0.76 *μ*g of NDPK-B to convert 43.78 *μ*moles of ATP per min) or 100 ng ml^−1^ VEGF (positive control) for 24 h. Cells were washed once with PBS and removed with trypsin (0.25%, 5 min) and counted using a Coulter cell counter (Beckman Coulter, Miami, FL, USA). Potential toxicity of EA was tested in HEC grown in a 48-well plate to 75% confluence with 10% FBS EGM media. The cells were grown in EBM-2 media containing 2% FBS for 24 h and then incubated with 10 *μ*M EA in 2% FBS EBM-2 media for 24 h. In some cultures, the medium containing 10 *μ*M EA was removed and the cells were washed with PBS and fixed with the Diff-quick Stain Kit (Polysciences, Warrington, PA, USA); in other cultures, the medium containing 10 *μ*M EA was removed, the cells were washed with 2% FBS EBM followed by addition of EGM containing 10% FBS and cultures incubated for another 24 h. Cells were then washed with PBS and fixed with the Diff-quick Stain Kit and effects of EA treatment determined by microscopy.

### Endothelial cell migration assay

The effects of NDPK-B, P2Y_1_R agonist and antagonist, and *Src* and VEGF antagonist on HEC migration were determined using a modified Boyden chamber assay. Human cord blood endothelial colony forming cells were cultured to ∼75% confluence on T-150 flasks and then switched to basal media (EBM-2) without growth factors supplemented with 2% FBS for 24 h. The serum-starved human endothelial cells (2.5 × 10^5^) were washed with PBS and then seeded onto the upper side of a Transwell insert (Corning, Lowell, MA, USA) membrane coated with a type-I rat-tail collagen (0.9 mg ml^−1^) and grown to 75% confluence. The agonist test compounds (100 ng ml^−1^ VEGF, 10 *μ*M 2MeS-ATP, 10 units NDPK-B, GTP/ADP alone; or 5–20 units NDPK-B with GTP/ADP) were added to the lower chamber, and antagonist compounds (10 *μ*M MRS2179, 50 *μ*M SU1498 and 100 nM PP2) were added to the upper chamber. Plates were then incubated for 24 h at 37°C, 5% CO_2_ in a humidified incubator. Following treatments, membranes were removed, residual cells in the upper chamber were scraped away, and the membrane stained with Diff-Quick solution (Dade Behring, Newark, DE, USA). Quantification was performed by counting dark blue nuclei in five contiguous microscopic fields ( × 60, centre, up, down, left and right) generating mean±s.e.m. cells per field. Micrographs were obtained at × 10 magnification.

### Statistical analyses

Graphs were prepared using Prism Graphing Software (V5; GraphPad Software, San Diego, CA, USA) and statistical analyses were performed using InStat Statistical Software (V3.0; GraphPad Software). All experiments were tested for statistical significance using ANOVA and a *P*⩽0.05 was considered significant. Data points and error bars represent mean values±s.e.m.

## Results

### Human breast carcinoma cell lines secrete human NDPK-B

**Figure 1:**
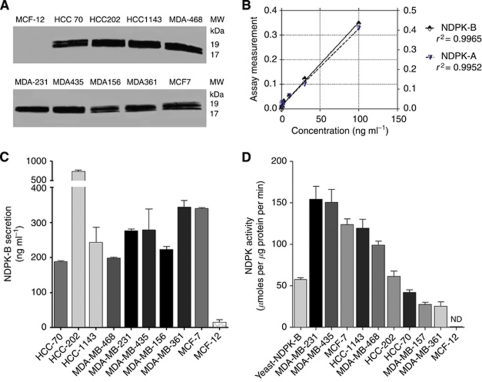
Detection of human NDPK-A/B protein secreted by breast cancer cell lines. Conditioned media from transformed and normal breast cell lines was collected after 1.5 h and concentrated as described in the text. (**A**) sNDPK-A/B protein was detected by western blot following separation of 25 *μ*g of total protein. (**B**) The concentration of sNDPK-A/B in conditioned media was quantified by ELSA assay. (**C**) Breast carcinoma cell lines secreted highly significant amounts of sNDPK. Data are mean±s.e.m., *n*=3. (**D**) Transphosphorylase activity was measured as ATP production in the luciferin-luciferase assay under *V*_max_ conditions (GTP 300 *μ*M, ADP 30 *μ*M). Yeast NDPK-B was employed as a positive control. Values, arrayed from high to low for comparison, are expressed as *μ*moles ATP produced per *μ*g protein per min. Data are mean±s.e.m., *n*=3 and are all highly significant compared with zero addition control.

We have shown previously that the human breast carcinoma cell line MDA-MB-435 secretes NDPK *in vitro* (Anzinger *et al*, 2001). The notion that NDPK secretion by these cells represented a unique adaptation to culture, a feature of this cell line alone, or the result of potential misidentification of the cells’ origins (Ross *et al*, 2000) undermined the hypothesis that NDPK secretion is a fundamental feature of transformed breast cells. We sought to further our hypothesis by examining additional cell lines derived from women with metastatic disease (Table 1) and known to be metastatic in murine models of breast cancer *in vivo* for extracellular NDPK activity that could support breast cell angiogenesis. western blot studies (Figure 1A; Table 1) revealed that breast cancer cell lines HCC70, HCC202, HCC1143, MDA-MB-468, MDA-MB-231, MDA-MB-435, MDA-MB-156, MDA-MB-361 and MCF-7 secreted shNDPK-A/B while we were able to detect only insignificant amounts of shNDPK from MCF-12 (a normal breast epithelial cell line) and detected no activity.

Next, we developed a sensitive and selective indirect ELISA for secreted human NDPK-A/B, which we employed to quantify sNDPK in conditioned medium from breast carcinoma cell lines. We found that breast carcinoma cell lines HCC70, HCC202, HCC1143, MDA-MB-468, MDA-MB-231, MDA-MB-435, MDA-MB-156, MDA-MB-361 and MCF-7 secreted significant amounts of sNDPK-A/B (187.8, 737.5, 243.0, 198.7, 275.6, 278.1, 221.9, 343.5 and 339.7 ng ml^−1^, respectively; Figures 1B and C), while none was detected in conditioned media from MCF-12 cells.

### sNDPK-B from breast cancer cell lines induces phosphorylation activity

We utilised a transphosphorylase assay to measure the ATP generating activity of sNDPK collected from the conditioned medium of 11 cell lines and compared it with the activity of purified yeast NDPK-B (Figure 1D). The assay was performed with GTP (phosphoryl donor), ADP (phosphoryl acceptor) and breast carcinoma cell conditioned medium or purified yeast NDPK-B under *V*_max_ conditions. Normal breast cells did not express extracellular NDPK activity, while ATP production from cancerous breast cell lines MDA-MB-231, MDA-MB-435, MCF-7, HCC1143, MDA-MB-468, HCC202, HCC70, MDA-MB-156, MDA-MB-361 and yeast NDPK-B (57.6 *μ*mol ATP per *μ*g protein per min) supported significant ATP production ranging from 154.4 to 25.58 *μ*mol ATP per *μ*g protein per min (Figure 1D). Cells that are known to form metastases *in vivo vs* those considered non-cancerous, confirmed that cells derived from women with metastatic disease secrete sNDPK (Table 1). Results were correlated with the activity of purified yeast NDPK-B and did not appear to correlate with expression of oestrogen receptor, or expression of the Her2 protein.

### Stimulation of P2Y receptors on HEC by NDPK and 2MeS-ATP induces VEGFR-2 activation

**Figure 2:**
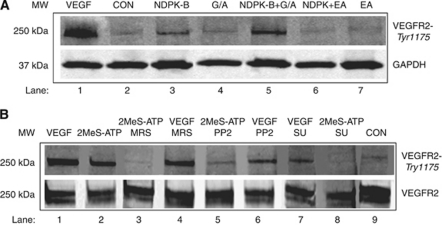
Extracellular NDPK-B and 2MeS-ATP activate VEGFR-2. Western blots carried out for the detection of activated VEGFR-2. HEC were maintained in low serum medium containing 2% FBS for 24 h before pretreatment with antagonoists followed by agonists. VEGF (100 ng ml^−1^, 10 min) employed as a positive control led to the phosphorylation of VEGFR-2 on tyrosine-1175 as expected (**A** and **B**). (**A**) Phosphorylated VEGFR-2 with loading control. (**B**) Tyr-1175 phosphorylation of VEGFR-2 and VEGFR-2 protein was blotted as a loading control. Data are representative of three experiments performed in duplicate.

Extracellular NDPK activity elevates ATP levels and activates endothelial P2Y receptors which in turn transactivate VEGFR-2, inducing the phosphorylation of VEGFR-2 Tyr-1175 (Rumjahn *et al*, 2009); an accepted indicator of the dimerisation and activation of VEGFR-2. Addition of VEGF_165_ (100 ng ml^−1^) to HEC cultures for 10 min led to VEGFR-2 Tyr-1175 phosphorylation compared with control cells receiving buffer alone (Figure 2A, lanes 1 and 2). The NDPK's enzymatic activity functions as an NDPK (Postel, 2003) regenerating ATP levels by covalently transferring the *γ*-phosphate from an NTP such as GTP to an NDP acceptor (e.g., ADP). We detected intense tyrosine 1175 phosphorylation of VEGFR-2 with addition of 10 units of NDPK in the presence of substrate and phosphoryl donor (Figure 2A, lane 5). Partial activation of VEGFR-2 (Tyr-1175 phosphorylation) by NDPK in the absence of exogenous GTP/ADP is consistent with the secretion of NDPK (Figure 2A, lane 3) as a phosphoryl-protein able to support one round of endogenous adenine trinucleotide formation (Anzinger *et al*, 2001). Our earlier work demonstrated that the catechins EGCG and EA inhibit cancer cell-secreted NDPK transphosphorylase activity (Rumjahn *et al*, 2007; Buxton, 2008). Ellagic acid is a more potent NDPK inhibitor than other known nucleoside analogues. Pretreatment of HEC cultures with EA (10 *μ*M) with and without NDPK-B (10 units) plus GTP/ADP diminished VEGFR-2 phosphorylation down to control levels (Figure 2A, lanes 6 and 7).

To further define the pathway mediating the effect of NDPK on VEGFR-2, we hypothesised that NDPK-regenerated ATP activates the endothelial purinergic nucleotide receptor (P2Y_1_R) resulting in *Src* phosphorylation which in turn phosphorylates VEGFR-2. Addition of the P2Y_1_R-specific agonist 2MeS-ATP led to VEGFR-2 Tyr-1175 phosphorylation (Figure 2B, lane 2) that was blocked by pretreatment of cells with P2Y_1_R, *Src* and VEGFR-2 selective antagonists lanes 3, 5 and 8, respectively (Figure 2B). Next, we compared the mechanism of P2Y_1_ mediated VEGFR-2 activation *vs* VEGF_165_ stimulation (100 ng ml^−1^) with and without the specific VEGFR-2 TRK inhibitor SU1498 employed as negative control. VEGF_165_ induced high VEGFR-2 phosphorylation levels and this activation was inhibited significantly by SU1498 (Figure 2B, lanes 1 and 7). MRS2179 (10 *μ*M) did not prevent the effect of VEGF_165_ (Figure 2B, lane 4). The *Src* inhibitor PP2 (100 nM) reduced, but did not block the effect of VEGF_165_ (Figure 2B, lane 6).

### Extracellular NDPK induces endothelial Erk_1/2_ phosphorylation

**Figure 3:**
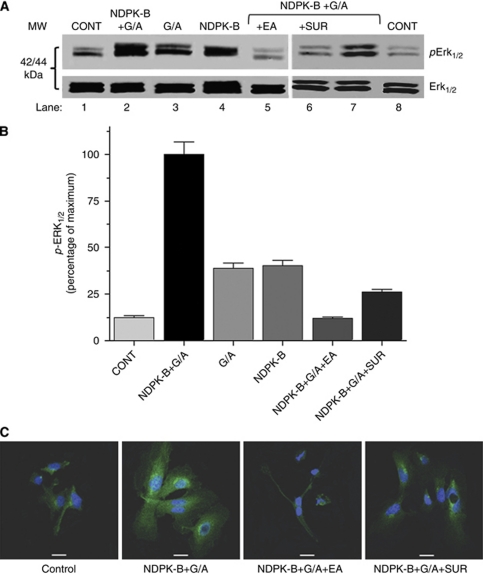
Extracellular NDPK-B induces HEC Erk_1/2_ phosphorylation. HEC were maintained in low serum medium containing 2% FBS for 24 h before pretreatment with antagonists followed by agonists. (**A**) Representative western blots showing both Erk and phospho-*Erk* (*pErk*_1/2_). (**B**) Average data from multiple experiments. All treatments gave significantly lower *pErk*_1/2_ (*P*<0.001) compared with addition of NDPK-B in the presence of phosphoryl donor and acceptor. Data are mean±s.e.m. normalised to total Erk expression by densitometry, *n*=3. (**C**) Representative confocal images of *pErk* fluorescence in HEC stimulated as shown (control 0.01% DMSO). (Bar=20 *μ*m).

Erk_1/2_ phosphorylation via the Ras-RAF-Erk pathway induces cell proliferation contributing to the activation of angiogenesis and metastatic tumour growth. We hypothesised that extracellular NDPK-B induces exogenous mitogenic signalling from endothelial P2Y_1_R to MAPK. Stimulation of HEC with NDPK-B in the presence of phosphoryl donor and acceptor produced high Erk_1/2_ phosphorylation (Figure 3A, lanes 2 and 7, B and C). With addition of NDPK-B or GTP/ADP alone, Erk_1/2_ phosphorylation was reduced by 60% compared with maximum stimulation (Figure 3B), indicating that they produced partial activation of Erk_1/2_ consistent with sub-maximal P2Y stimulation. The effect of NDPK-B was inhibited by 10 *μ*M EA (Figure 3A, lane 5) which diminished Erk_1/2_ phosphorylation back to near control levels (Figure 3). VEGF_165_ was employed as positive control and gave a 11-fold increase above the negative control (data not shown). Suramin (100 *μ*M), a non-specific endothelial P2Y receptor inhibitor (Beindl *et al*, 1996), blocked 70% of the effect of 10 units NDPK-B plus donor/acceptor (Figure 3). Measurement of Erk_1/2_ phosphorylation by immunofluorescence confirmed that extracellular NDPK-B induced Erk_1/2_ phosphorylation, which was inhibited by the NDPK transphosphorylation inhibitor (EA) and non-specific P2Y receptor inhibitor suramin (Figure 3C).

### Activated P2Y_1_R induces Erk phosphorylation

**Figure 4:**
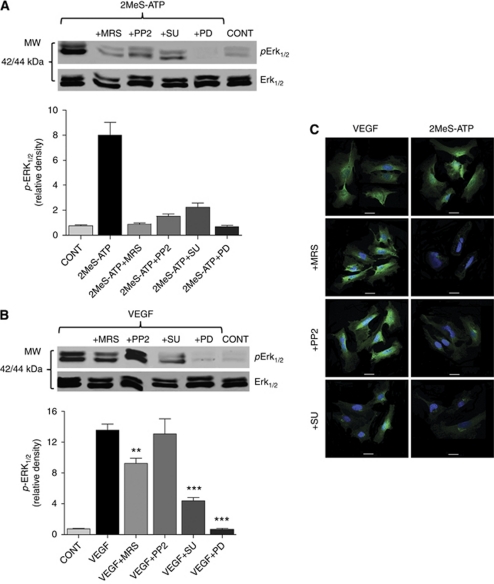
Erk_1/2_ phosphorylation by P2Y_1_R agonist or VEGF. HEC were maintained in low serum medium containing 2% FBS for 24 h before treatment with (**A**) 2MeS-ATP (10 *μ*M) or (**B**) VEGF (100 ng ml^−1^) following antagonist pretreatment. (**A**) 2MeS-ATP produced significant activation of *Erk*_1/2_ that was significantly blocked (*P*<0.001) by MRS2179, PP2, SU1498 or PD89059. (**B**) VEGF activation of *Erk*_1/2_ showed partial significant sensitivity to MRS2179, but insensitivity to PP2. Control (CONT) received 0.01% DMSO as a drug dilution control. (**A** and **B**) Data are mean±s.e.m. normalised to total Erk expression by densitometry; one-fold=non-stimulated control, *n*=3. (**C**) Imaging of phospho-*Erk*_1/2_; cells were pretreated with 10 *μ*M MRS2179, 100 nM PP2 or 50 *μ*M SU1498. Images are representative of multiple micrographs aquired from three experiemnts; bar=20 *μ*m.

To further assess the potential mechanism of exogenous mitogenic signalling from P2Y_1_R to mitogen-activated MAPK in endothelial angiogenesis, we determined the effect of P2Y_1_R (MRS2179), *Src* (PP2), VEGFR-2 (SU1498) and MEK (PD98059) antagonists on nucleotide signalling. The relative magnitude of the effect of Erk_1/2_ MAPK in mediating the actions of the endothelial nucleotide receptors can be seen when comparing the effect of the MAPK inhibitor PD98059 on the activation of Erk_1/2_ when HEC are stimulated by either 10 *μ*M 2MeS-ATP (Figures 4A and C) or 100 ng ml^−1^ VEGF_165_ (Figures 4B and C). In both cases, the agonist effects are completely blocked by PD98059. P2Y_1_R activation by 2MeS-ATP induced Erk_1/2_ phosphorylation that was inhibited by MRS2179 (87.5%), PP2 (77.5%), SU1498 (73.7%) or PD98059 (90%), respectively (Figure 4A). VEGF_165_-mediated Erk_1/2_ phosphorylation was inhibited only 33% by MRS2179 (Figure 4B). Our western blot and immunofluorescence results indicate that 100 nM PP2 (Figures 4B and C) has no effect on VEGF_165_-stimulated Erk_1/2_ phosphorylation. SU1498 inhibited VEGFR-2 phosphorylation (68%). The ability of the VEGFR-2 antagonist SU1498 (50 *μ*M) to prevent Erk_1/2_ activation by either VEGFR-2 or P2Y_1_ receptor stimulation demonstrates that P2Y_1_ endothelial receptors signal through VEGFR-2 (Figure 4).

### Effects of NDPK-B on endothelial cell proliferation

**Figure 5:**
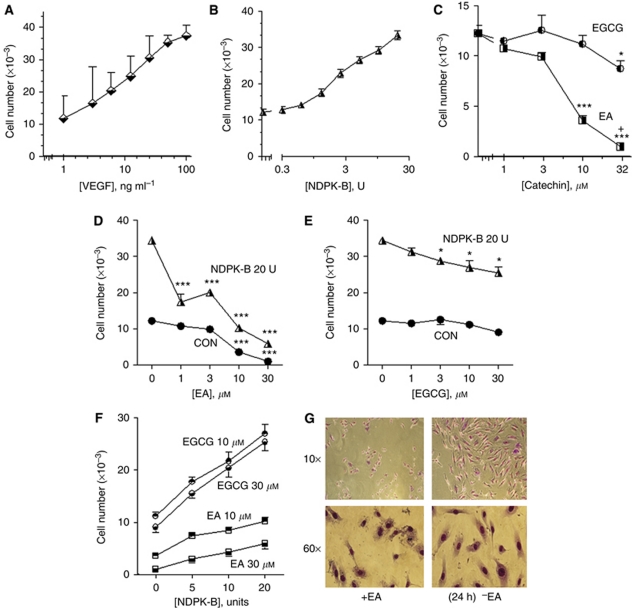
Extracellular NDPK-B stimulates HEC growth and NDPK-B transphosphorylation inhibitors block the growth stimulatory effects of NDPK-B. HEC were maintained in low serum medium containing 2% FBS for 24 h before treatment with increasing concentrations of (**A**) VEGF, (**B**) NDPK-B or (**C**) catechin (EA/EGCG) for an additional 24 h in 2% FBS containing 0.01% (v/v) DMSO diluent in EBM media. (**A**) VEGF protein and (**B**) NDPK-B protein (**C**) treatment of cells with the NDPK-B antagonist catechins EA or EGCG at increasing concentrations. (**D**) Twenty units of NDPK-B in combination with increasing concentrations of EA. (**E**) Twenty units of NDPK-B in combination with increasing concentrations of EGCG. (**F**) Increasing concentrations of NDPK-B in combination with 10 or 30 *μ*M concentrations of EGCG or EA. (**G**) The drug is removed and cells are returned to growth conditions. Data are mean±s.e.m., *n*=3, *P<0.05, ^***^P<0.001 *vs* no drug, ^+^*P*<0.05 *vs* 10 *μ*M EA. Micrographs are representative of results, *n*=4.

Secreted NDPK-B induces angiogenesis as measured by endothelial cell tubule-like formation (Rumjahn *et al*, 2007). Here, we further investigated the role of NDPK-B in endothelial angiogenesis. We hypothesised that the activation of P2Y_1_R by extracellular NDPK-B would promote the growth of endothelial cells *in vitro*, and that inhibiting the function of NDPK-B would reduce endothelial cell proliferation. Human cord blood endothelial colony forming cells were stimulated for 24 h with 1–100 ng/ml VEGF (positive control) or 0.3–20 units of NDPK-B. Extracellular NDPK-B was as effective as VEGF in stimulating HEC proliferation (Figures 5A and B) over the 24-h period. The concentration range of NDPK employed here (0.3–30 units=0.5–500 ng ml^−1^) correlates well with the activity of sNDPK measured in breast cancer cell conditioned media (Ave. 272 ng ml^−1^). If the effect of NDPK-B relies on ATP generation, catechin compounds known to inhibit NDPK activity would prevent NDPK effects on cell proliferation (Figure 5C). Ellagic acid was more effective than EGCG in inhibiting cell growth consistent with its effect as an NDPK inhibitor (Buxton, 2008) and blocker of the effect of NDPK to promote angiogenesis (Rumjahn *et al*, 2007). To examine the effect of extracellular NDPK-B protein and its inhibitors in primary HEC proliferation, HEC were incubated with various concentrations of EA and EGCG (1–30 *μ*M) with and without 20 units of NDPK-B protein for 24 h (Figures 5D and E). The activity of NDPK-B to induce angiogenesis via cell proliferation was diminished to the basal level in the presence of 10 *μ*M EA (Figure 5D). The results indicate that 10 and 30 *μ*M EGCG inhibit HEC proliferation (Figures 5C and E), but when combined with 20 units of extracellular NDPK-B, EGCG inhibition is trivial (Figure 5E). Cells were also treated with various amounts of NDPK-B (0, 5, 10 and 20 units) in the presence of EGCG or EA (10 or 30 *μ*M) (Figure 5F). The effects of EA and EGCG were dose dependent (Figures 5D and E) and confirmed that EA is more efficacious than EGCG in preventing NDPK-mediated cell proliferation (Figures 5C–F). Control experiments confirmed that EA was non-toxic to the cells and show that the drug acts in a reversible manner (Figure 5G).

### Extracellular NDPK-B induces ECFC migration

**Figure 6:**
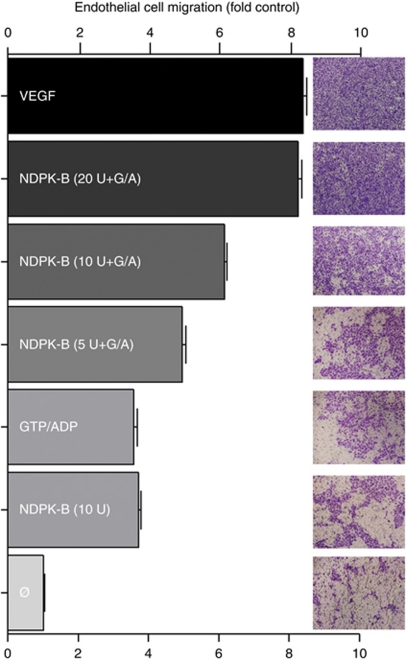
Extracellular NDPK-B stimulated HEC migration. HEC were maintained in low serum medium for 24 h. HEC growing on membrane supports coated with collagen were stimulated from below for 24 h with VEGF (100 ng ml^−1^) as positive control, or by increasing concentrations of NDPK-B in the presence of GTP 300 *μ*M/ADP 30 *μ*M, control sets were GTP/ADP, 10 units NDPK-B alone and negative control (Ø) in 2% FBS in EBM media. Membranes were prepared and stained as described in the text. Data are mean±s.e.m. in 3–5 experiments. Data are expressed as fold control where 1=no addition (Ø). All treatments produced significant migration compared with control (*P*<0.03).

We determined the effect of P2Y_1_R activation by extracellular NDPK-B protein on HEC migration *in vitro* using a modified Boyden chamber assay. VEGF was employed as positive control. The NDPK-B protein was added at various concentrations (5, 10 and 20 units; Figure 6) and cells stimulated for 24 h. Migration stimulated by VEGF (100 ng ml^−1^) was matched by treatment with 20 units of NDPK-B in the presence of phosphoryl donor and acceptor (GTP, 300 *μ*M; ADP, 30 *μ*M). The effect of NDPK-B was dose dependent (Figure 6). The phosphoryl donor acceptor mix (GTP+ADP) and NDPK-B alone had an effect *vs* control (no addition) consistent with the notion that ADP alone can activate P2Y receptors and that NDPK-B alone has been shown to stimulate angiogenesis (Rumjahn *et al*, 2007).

**Figure 7:**
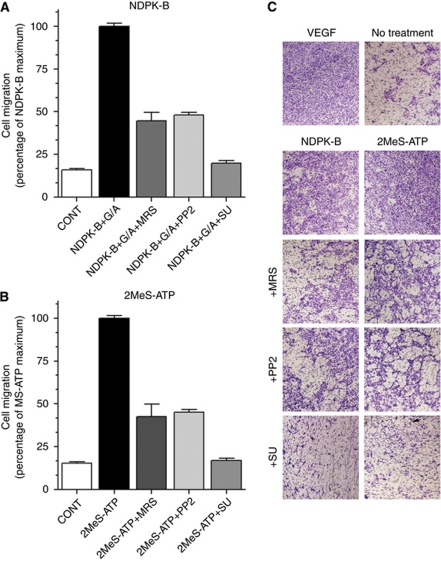
Antagonism of 2MeS-ATP or NDPK-B-mediated HEC migration is consistent with Src activation of VEGFR-2. HEC were seeded on the upper surface of collagen-coated membranes as described in the text. (**A**) Ten units of NDPK-B added in the presence of phosphoryl donor and acceptor that produced *V*_max_ conditions for ATP production. (**B**). All antagonist treatments (**A** and **B**) produced highly significant blockade. (**C**) Micrographs representative of the result in each experiment. Membranes were prepared and stained as described; migrated cells were counted and values expressed as percentage of the maximum stimulation defined as VEGF-stimulated migration. Values are mean±s.e.m., *n*=3–5 and all statistical comparisons of the effects of inhibitors on stimulated migration (**A** and **B**) are significant at *P*<0.001.

We determined the effect of extracellular NDPK-B *vs* specific P2Y_1_ activation by 2MeS-ATP on endothelial cell migration using a modified Boyden chamber assay. Activation of P2Y_1_R by 2MeS-ATP or extracellular NDPK-B is as effective as VEGF in stimulating endothelial cell migration (Figure 7C). Stimulation of HEC by either 10 *μ*M 2MeS-ATP or 10 units of NDPK-B in the presence of GTP and ADP induced similar levels of migration (∼7-fold above the negative control, Figure 7). Human cord blood endothelial colony forming cells migration stimulated by 2MeS-ATP or NDPK-B plus GTP/ADP was significantly reduced by the P2Y_1_R antagonist MRS2179 or the *Src* inhibitor PP2 and to the same extent, while the VEGFR-2 antagonist SU1498 (Figure 7) was far more effective blocking essentially all of the stimulation produced by either agonist.

## Discussion

ATP is released by endothelial cells under sheer stress (Milner *et al*, 1996) and hypoxic conditions and is both vasodilatory (Yang *et al*, 1994; Buxton *et al*, 2001) and pro-angiogenic (Gerasimovskaya *et al*, 2008). Here, we offer evidence of the pathologic ability of breast tumour cells to stimulate endothelial angiogenesis. We found that a panel of cancerous human breast cell lines, representing many of the models used in recent years by investigators studying the metastatic process, secreted both NDPK-A/B while normal cultured breast cell lines do not. We detected an ∼19-kDa band (NDPK-A) and ∼17-kDa band (NDPK-B) by western blot and quantified them by ELISA (Figures 1A and C). We showed that the ELISA developed with anti-NDPK-B antibody also recognises the NDPK-A protein (Figure 1B). Nucleoside diphosphate kinase-A has been found in neuroblastoma (Okabe-Kado *et al*, 2005b), B and T-cell lymphoma (Niitsu *et al*, 2003, 2004), haematological malignancies (Okabe-Kado, 2002) and leukaemia (Niitsu *et al*, 2000; Okabe-Kado *et al*, 2002) where it induces cell proliferation (Okabe-Kado *et al*, 2009b) and activates cytokine production (Okabe-Kado *et al*, 2009a).

**Figure 8:**
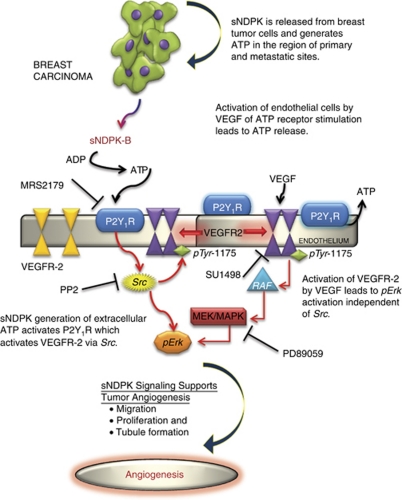
Endothelial nucleotide receptor signalling in tumour angiogenesis. VEGFR-2, the principle receptor on endothelium known to regulate angiogenesis is activated by VEGF. We demonstrate here that the endothelial nucleotide P2Y receptor is an important angiogenesis regulator. In the tumour micro-environment, tumour cells secrete sNDPK which generates ATP from ADP locally. ADP availability is predicted from necrosing cells and acts on the endothelial nucleotide P2Y receptor to regulate blood flow and vascular permeability, which in turn enables the metastatic process. Our results reveal that sNDPK release by breast tumour cells activates VEGFR-2 via P2Y_1_R stimulation. Inhibition of P2Y_1_R, *Src* and VEGFR-2 tyrosine kinase phosphorylation prevents extracellular NDPK-B/2MeS-ATP from inducing *Erk/*MAPK activation and cell migration. Therefore, we propose that P2Y_1_R receptor activation could be an early signal to activate growth of dormant metastases that do not themselves as yet secrete VEGF. Considerable focus should be placed on nucleotide signalling in angiogenesis as this pathway may become a therapeutic target for tumour anti-angiogenesis in breast cancer.

Our results demonstrated conclusively that the effects of endothelial P2Y_1_ receptor stimulation result in the activation of VEGFR-2 in the absence of VEGF. This striking result is borne out by the ability of the VEGFR-2 antagonist SU1498 to block the effect of P2Y receptor agonist to cause VEGFR-2 phosphorylation (Figure 2B). The ability of breast tumour cells to orchestrate angiogenesis is provided for by their ability to secrete NDPK, which can result in transactivation of the VEGF receptor-2 even in the absence of VEGF (Figure 2A). The enhancement of the effect of NDPK-B when a phosphoryl donor and substrate acceptor are present, together with the ability of the NDPK inhibitor EA to block VEGFR-2 phosphorylation establishes the effect of NDPK-B as acting through P2Y receptor stimulation. The ability of 2MeS-ATP to mimic the effect of NDPK-B, and for both to be prevented by the P2Y_1_R antagonist MRS2179 is convincing proof that NDPK-B acts through P2Y_1_R activation in HEC. The fact that NDPK-B activates its vascular endothelial receptor (P2Y receptor), which in turn activates VEGFR-2 in the absence of VEGF (Figures 2 and 8) offers the intriguing possibility that focusing on P2Y mechanisms in angiogenesis might broaden the armamentarium in prevention of breast cancer metastasis.

Endothelial cell VEGFR-2 activation by extracellular NDPK-B results in *Erk*_1/2_ phosphorylation, which is prevented by EA, an NDPK inhibitor, and suramin a non-specific P2YR antagonist (Figure 3). Together, these results indicate that activation of P2Y receptors by extracellular NDPK-B is crucial in the transactivation of VEGFR-2 and subsequent downstream regulation of the Raf-MEK-MAPK pathway (Figure 8).

The ability of the *Src* inhibitor PP2 to block both P2Y_1_R and VEGF stimulation of VEGFR-2 phosphorylation (Figure 2B), while having little effect on VEGF-stimulated *Erk*_1/2_ phosphorylation is consistent with the notion that P2Y_1_R signals to activate VEGFR-2 via *Src* activation (Figure 4B). The ability of VEGF to activate VEGFR-2 Tyr-1175 phosphorylation is, by contrast, a direct effect of the growth factor to bind and activate its receptor (Figure 2B). The contrast in the transactivation of VEGFR-2 by P2Y_1_R activation *vs* VEGF activation can be seen in the ability of PP2 to block P2Y_1_R but not VEGF activation of VEGFR-2 (Figure 2B). PP2 fails to prevent VEGF activation of *Erk*_1/2_ (Figure 4B), confirming that Src does not phosphorylate *Erk* in human endothelial cells.

We have previously shown that the activation of the P2Y_1_ receptor is crucial in the activation of tubule formation in endothelial cells (Rumjahn *et al*, 2007). In this study, we were particularly interested in examining the mechanisms of the P2Y_1_ receptor pathway with regard to transactivation of VEGFR-2 Tyr-1175, *Erk*_1/2_ activation, and functional determination of cell growth and migration (Figures 5, 6 and 7). The ability of VEGF to stimulate endothelial cell growth is mimicked by NDPK-B in a concentration-dependent manner (Figure 5). Endothelial cell proliferation is prevented by EA and partially effected by EGCG, which is consistent with the idea that these compounds block NDPK-B activity. While EA treatment prevents endothelial proliferation induced by NDPK-B, it is not cytotoxic and the cells recover after removal of EA (Figure 5G). VEGF is known to promote endothelial cell migration as well as cell proliferation. We examined the effects of both NDPK-B and VEGF on endothelial cell migration and found that 20 units of NDPK stimulated endothelial cell migration that was equivalent to 100 ng VEGF under the same conditions and the effect of NDPK was concentration dependent (Figure 6).

In examining the mechanism of action of sNDPK, it is interesting to note that sNDPK is thought to bind to the high molecular weight fragment of MUC-1 (Mahanta *et al*, 2008), also shown to be present in the conditioned medium from breast cancer cells in culture (Thathiah *et al*, 2003). The finding that purified bovine NDPK binds to MUC-1 was seen as evidence for its growth stimulatory properties in human embryonic stem cells (Hikita *et al*, 2008). In the studies described here and elsewhere (Buxton *et al*, 2010), NDPK is acting via P2Y receptor activation in an ATP-dependent manner. While we do not rule out a role for the MUC-1 protein in the breast cancer angiogenic process; indeed it would mediate actions of NDPK acting directly and as such fits with our overall hypothesis regarding the importance of sNDPK; the effects of sNDPK we have measured are best explained by its action as a generator of purinergic agonist. Future studies to link MUC-1 and sNDPK and possibly the P2 receptor may further our understanding of tumour cell-mediated angiogenesis.

The extracellular actions of purine nucleotides are known to be important in bleeding disorders, hypertension, stroke and osteoporosis, and it is also important for physiological functions such as vasodilation, platelet aggregation, and neural and stem cell proliferation. Here, we demonstrated that the P2Y_1_R regulates endothelial migration following the action of soluble NDPK-B or 2MeS-ATP and that activation was blocked by the P2Y_1_R receptor antagonist (MRS2179) as well as the *Src* kinase blocker PP2 (Figure 7). The *Src* family kinases are signalling enzymes that have been recognised as cellular process regulators, and it is known that *Src* has an important role in the promotion of endothelial monolayer permeability (Hu *et al*, 2008) and in supporting FAK signalling in VEGF-induced endothelial cell migration and survival (Abu-Ghazaleh *et al*, 2001). The ability of the VEGFR-2 antagonist SU1498 to block both NDPK-B and 2MeS-ATP effects on cell migration confirmed that these effects are mediated by VEGFR-2 (Figure 7).

A role for nucleotides in the angiogenic process in this manner suggests potential therapeutic targets that might help to control latency. However, the mechanisms by which P2Y receptors mediate vasodilation and anti-platelet aggregation (advantageous to the transit of cancer cells to secondary sites), and tumour angiogenesis remains to be defined *in vivo*. Because endothelial P2Y_1_ receptors are thought to participate in transendothelial migration (Sud’ina *et al*, 1998), we propose that, in addition to the notion that tumour-secreted sNDPK promotes endothelial cell growth and migration in support of angiogenesis at the metastatic niche, release of sNDPK during early primary tumour development can promote transendothelial cell migration and movement of cells out of the breast to distant sites in the body (Figure 8).

## Figures and Tables

**Table 1:** Breast cell NDPK-secretion correlates with cancerous origin

**Cell line**	**ATCC #**	**Metastatic**	**ER** **^+^**	**Her2^+^**	**shNDPK-B secretion**	
*Cell line grown in culture*						
					
HCC70	CRL-2315	Yes-DC-primary	Yes	No	Yes [++]	
HCC202	CRL-2316	Yes-DC-primary	No	Yes	Yes [++]	
MDA-MB-468	HTB-132	Yes-AC-plural effusion	No	Yes	Yes [++}	
MDA-MB-361	HTB-27	Yes-AC-brain	Yes	Yes	Yes [+]	
MDA-MB-157	HTB-24	Yes-MC-plural effusion	No	unk	Yes [+]	
MDA-MB-435S^a^	HTB-129	Yes-DC-plural effusion	No	Yes	Yes [+++]	
MDA-MB-231	HTB-26	Yes-AC-plural effusion	No	No	Yes [+++]	
HCC1143	CRL-2321	Yes-DC-plural effusion	No	No	Yes [+++]	
MCF7	HTB-22	Yes-AC-plural effusion	Yes	Yes	Yes [+++]	
MCF-12A	CRL-10782	No-normal mammary epithelium (immortal)	Yes	No	No [−]	
MCF-10A	CRL-10317	No-fibrocystic disease, epithelium	No	No	No [−]	
					
**Mice**	**Cells implanted**	**Location of implant**	**Number implanted**	**Time after implant**	**Serum NDPK (ng ml^−1^)**	
					**Control**	**Tumours**
*NDPK measured in vivo*						
						
SCID	MDA-MB-231-Luc+ 2.5 × 10^6^	Mammary fat	6	4 Weeks	27.8	260.9
			10	8 Weeks	26.5	250.0

Abbreviations: AC=adenocarcinoma; DC=ductal carcinoma; ER=estrogen receptor; MC=medullar carcinoma; NDPK=nucleoside diphosphate kinase; unk=unknown.

a Gene expression analysis of MDA-MB-435S produced microarrays in which these cells clustered with cell lines of melanoma origin instead of breast (Rae *et al*, 2004).

[+]Positivity related to control using purified yeast NDPK-B (57.6±3.8 *μ*moles ATP per *μ*g per min) under *V*_max_ conditions as [++].
